# Ultrasound segmentation analysis via distinct and completed anatomical borders

**DOI:** 10.1007/s11548-024-03170-7

**Published:** 2024-05-25

**Authors:** Vanessa Gonzalez Duque, Alexandra Marquardt, Yordanka Velikova, Lilian Lacourpaille, Antoine Nordez, Marion Crouzier, Hong Joo Lee, Diana Mateus, Nassir Navab

**Affiliations:** 1 Computer-Aided Medical Procedure and Augmented Reality (CAMP), CIT, Technical University of Munich, Garching bei Muenchen, Germany; 2 Munich Center for Machine Learning, Munich, Germany; 3 LS2N Laboratory, Ecole Centrale Nantes, Nantes, France; 4 MIP Laboratory, EA 4334, 44000 Nantes, France

**Keywords:** Ultrasound segmentation, Anatomical borders, 3D US dataset

## Abstract

****Purpose**:**

Segmenting ultrasound images is important for precise area and/or volume calculations, ensuring reliable diagnosis and effective treatment evaluation for diseases. Recently, many segmentation methods have been proposed and shown impressive performance. However, currently, there is no deeper understanding of how networks segment target regions or how they define the boundaries. In this paper, we present a new approach that analyzes ultrasound segmentation networks in terms of learned borders because border delimitation is challenging in ultrasound.

****Methods**:**

We propose a way to split the boundaries for ultrasound images into distinct and completed. By exploiting the Grad-CAM of the split borders, we analyze the areas each network pays attention to. Further, we calculate the ratio of correct predictions for distinct and completed borders. We conducted experiments on an in-house leg ultrasound dataset (LEG-3D-US) as well as on two additional public datasets of thyroid, nerves, and one private for prostate.

****Results**:**

Quantitatively, the networks exhibit around 10% improvement in handling completed borders compared to distinct borders. Similar to doctors, the network struggles to define the borders in less visible areas. Additionally, the Seg-Grad-CAM analysis underscores how completion uses distinct borders and landmarks, while distinct focuses mainly on the shiny structures. We also observe variations depending on the attention mechanism of each architecture.

****Conclusion**:**

In this work, we highlight the importance of studying ultrasound borders differently than other modalities such as MRI or CT. We split the borders into distinct and completed, similar to clinicians, and show the quality of the network-learned information for these two types of borders. Additionally, we open-source a 3D leg ultrasound dataset to the community https://github.com/Al3xand1a/segmentation-border-analysis.

**Supplementary Information:**

The online version contains supplementary material available at 10.1007/s11548-024-03170-7.

## Introduction

Ultrasound (US) imaging is essential in medical diagnostics, providing non-invasive visualization of internal organs. Its advantages include versatility, safety, and portability, making it a first choice in fields like obstetrics, cardiology, and oncology [1]. Segmenting specific regions in ultrasound aids in diagnosing medical conditions by analyzing organs like the liver, heart, or kidneys. However, segmenting ultrasound images can be challenging due to factors like noise, speckles, shadows, and low contrast [2]. Unlike CT or MRI, which show consistent tissue distributions, ultrasound depends on the absorption and reflection of acoustic waves at tissue interfaces. As a result, delineation of anatomical boundaries can be particularly challenging in regions exhibiting low signal intensity or in the presence of artifacts.

Many medical practitioners tackle US segmentation challenges by first classifying, then focusing on a region of interest, and finally separating structures based on tissue interfaces [3]. Specifically, during ultrasound segmentation, delineations are drawn along distinct boundaries in clear regions. Subsequently, in regions with less clarity, completion is performed, guided by prior knowledge and experience.

As ultrasound is a dynamic imaging method, it requires anatomical and positional context for accurate localization of structures. Therefore, evaluating segmentation methods, and in particular neural network performance for ultrasound image segmentation, should account for these complexities. As a result, we suggest evaluating separately the ability to delineate the clear regions and the ability to complete the unclear boundary regions.

Despite its importance, most neural networks for ultrasound segmentation are assessed by comparing one expert label with the prediction as a learning signal. Metrics like Dice score or Hausdorff distance rank networks but don’t necessarily reflect the network’s understanding of border information. Particularly in ultrasound imaging, where borders are often ambiguous, it is important to understand the information that the networks rely on for segmentation to ensure a robust evaluation.

Recent works proposed Grad-CAM [4, 5] and other interpretation methods [6, 7] to understand what networks look at when segmenting medical images. Most of the work mainly focus on MRI or CT and a few on ultrasound [8, 9], evaluating the activation for complete structures.

In this paper, we concentrate on assessing networks based on their learned representation within border regions rather than solely evaluating the accuracy of the predictions. Specifically, we distinguish between distinct and completed boundaries, aiming to emulate the techniques of medical practitioners. In our study, we conduct comprehensive evaluations across four ultrasound datasets for three encoder-decoder style architectures [10–12]. Additionally, we open-source a LEG-3D-US dataset containing segmentation at different depth levels, even in areas where doctors interpolate. Thus, we facilitate the repeatability of the experiments and contribute to annotated medical datasets. In summary, our contribution can be summarized as follows:

1. We present a novel approach for analyzing ultrasound segmentation networks, distinguishing between distinct and completed borders, and mirroring medical practitioners’ evaluations.
2. We exploit the Grad-CAM to highlight the specific regions prioritized by each segmentation network across four diverse ultrasound datasets.
3. We make publicly available a LEG-3D-US dataset, facilitating further research and ensuring reproducibility.

## Related work

### Evaluating learned information

While several methods have been developed for enhanced model interpretation [13, 14], Gradient-weighted Class Activation Mapping (Grad-CAM) has emerged as a prominent tool. Initially introduced for classification in [4], Grad-CAM highlights regions of an input image that significantly influence a model’s prediction, providing insights into why a model made a specific decision. Many works have employed Grad-CAM to evaluate the learned information of classification networks [4, 5]. When it is evaluated on medical images, its task is to explain the decision-making processes for architectures’ comprehension. However, to the best of our knowledge, there are no qualitative Grad-CAM evaluations of ultrasound borders. It is a crucial task to enable reliable diagnoses and effective treatment planning [15], for tumor localization [16], lesion detection [17], or segmentation [18, 19], in a qualitative manner. Hence, in this paper, we aim to analyze network activations for borders, particularly differentiating between distinct and completed types, utilizing Seg-Grad-CAM as an explainability tool.

### Open-source ultrasound datasets

Segmenting ultrasound images using neural networks requires high-quality datasets because of their complexity and difficulty. These datasets, standardized by specific acquisition protocols, should be made openly available to encourage innovation and collaboration within the medical imaging community. Open-source datasets provide a standardized benchmark, enabling researchers globally to train, test, and refine their algorithms, ensuring more rapid advancement of diagnostic tools. Moreover, it democratizes access and allows researchers from varied backgrounds to contribute, leading to diverse and robust models that cover a broader range of medical conditions and patient demographics.

In this section, we briefly describe previously released open-source datasets [20, 21] and one internal 3D prostate dataset. Four ultrasound datasets for thyroid, nerve, leg, and prostate segmentation have been observed in this work. Among them, two have been previously released open-source datasets [20, 21], and one is a private 3D prostate dataset. Additionally, a 3D leg ultrasound dataset (LEG-3D-US) was collected and will be made open-source. Different from the previous datasets, with relatively homogeneous images as the thyroid and prostate datasets, the Leg-3D-dataset contains muscles that are not homogeneous tissues, suffer from blurred and hard-to-delineate borders, and splits segmentation of muscles in 3 groups, which is more challenging compared with a vein vs a muscle segmentation. It could be very interesting to try our method in other more challenging datasets such as kidney, brain, or fetal anatomy, if such datasets were made open-source, for now, we encourage researchers to try our method on their datasets. Following are additional details on the datasets.

*Thyroid dataset:* Kronke et al. [20] provided 32 volumes of 16 participants of the left and the right sides of the neck. With 3 labels segmented: the thyroid, the jugular vein, and the carotid artery. 3D volumes with pixel resolution of 380 $\times$ 330 $\times$ 300 and voxel spacing of 0.12mm were acquired with a 3D curvilinear probe of 64 channels.

*Nerve-UTP-2D dataset:* Jimenez et al. [21] provides 691 2D ultrasound images of nerves sized $360 \times 279$ pixels, cropped from original $640 \times 480$ images, with nerves covering 30% of the area. Captured using a SONOSITE Nano-Max device, it includes images of the sciatic nerve (287 instances), ulnar nerve (221 instances), median nerve (41 instances), and femoral nerve (70 instances). They use a linear 4–16MHz probe.

*Prostate 3D dataset:* It is an internal dataset of 40 patients with suspected cancer, 3D volumes with a pixel resolution of $230 \times 230 \times 70$ and voxel spacing of 0.27 mm. For each participant was performed an ultrasound and a magnetic resonance-T1 (MRI) acquisition. Ultrasound volumes were recorded with a rectal ultrasound probe Wisonic Endokavitär Sonde EV10-4 using the Acuson Juniper Siemens ultrasound machine. Labels of the prostate were transferred from annotations done in the MRI images after registration of the ultrasound volumes.

## Open-source LEG-3D-US dataset

**Fig. 1:**
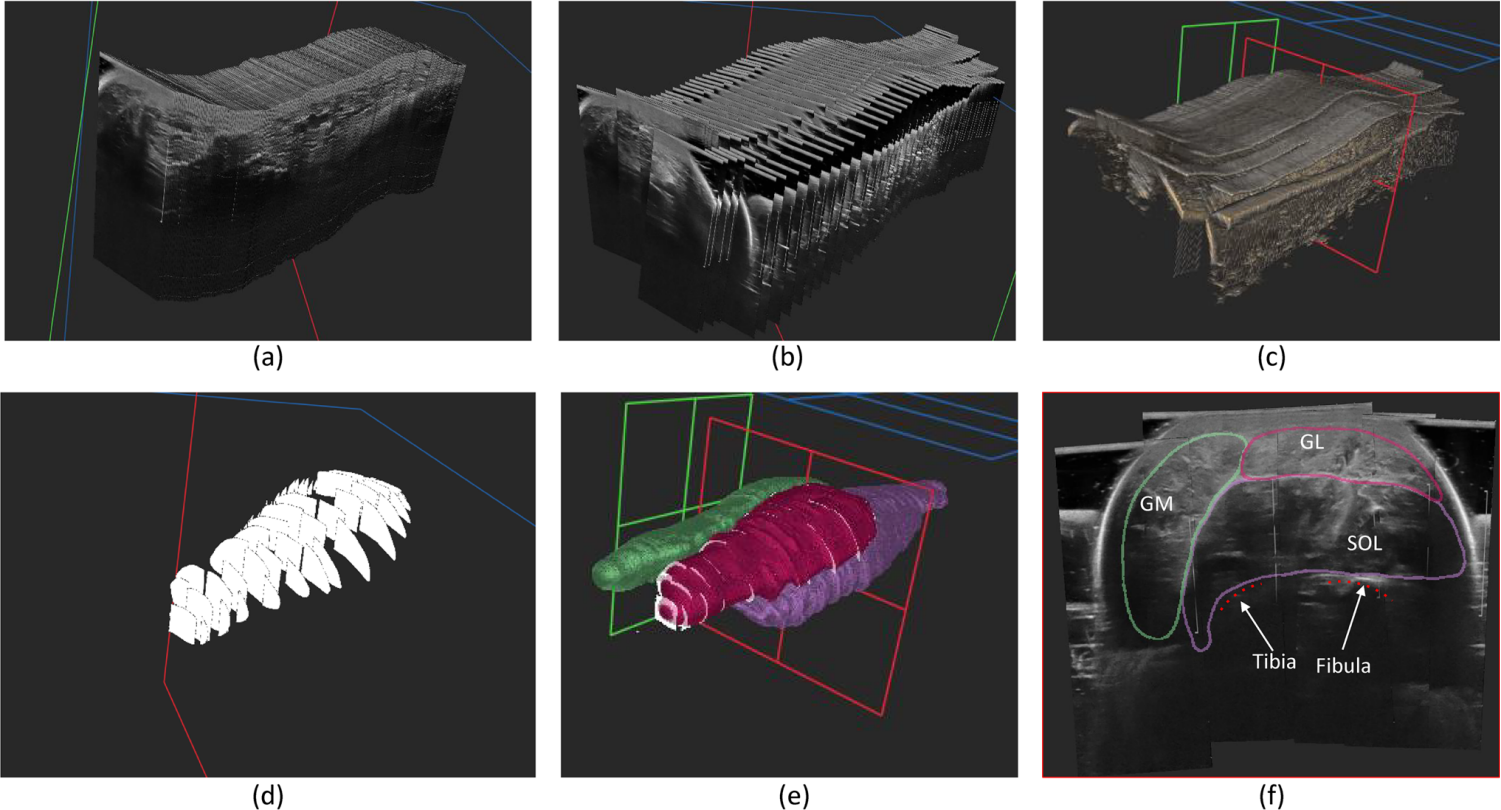
LEG-3D-US dataset: **a** Single ultrasound sweep, **b** 5 sweeps, **c** 3D volumes reconstructed, **d** Sparse annotations **e** Interpolations **f** Cross-sectional view soleus (SOL), Gastrocnemius Lateralis (GL), Gastrocnemius Medialis (GM)

The LEG-3D-US dataset contains 44 ultrasound volumes from legs of participants aged 18–45. It is part of the Achilles tendinopathy study by Crouzier et al. [22], with ethical approval of the local ethics committee (Rennes Ouest V-CPP-MIP-010) with all procedures adhered to the declaration of Helsinki. Images were acquired with the participant’s leg in a custom water tank (Fig. 1a) to avoid muscle deformation due to probe pressure. Freehand ultrasound with six optical tracking camera systems was used (Optitrack, Natural point, USA) for acquiring the 3D volumes. The acquisition took images from knee to ankle, in 4–6 overlapping sweeps (Fig. 1b) using a 50-mm linear probe (4–15 MHz; Aixplorer, Supersonic Imagine, Aix-en-Provence, France). Volumes were created using the compound volume algorithm in ImFusion Suite software,^1^ with a Gaussian kernel of 5 pixels (Fig. 1c). Each volume consists of a voxel grid with size $564 \times 632 \times 1443\pm (49\times 38\times 207)$ and an average spacing 179 of 0.276993 mm$^3 \pm 0.0015$ mm$^3$.

Sparse muscle annotations were performed in 2D B-mode high-resolution images on the Stradwin Software [23] by 2 annotators with an intra-operator error of 4%. Annotators segmented gastrocnemius medialis, gastrocnemius lateralis, and soleus muscles. 3D-muscle models were reconstructed with the “ZOI method” [3] and for 15 of the participants, additional refinement was done by one of the experts. We make the LEG-3D-US dataset publicly available.

## Proposed clinician-inspired evaluation

The core of our method is to evaluate segmentation networks using Seg-Grad-CAM in completed and distinct borders. This split is crucial to understanding the expert’s annotation process. Different from CT and MRI which contain regions with similar intensities, ultrasound intensities are not uniform because of the sound’s interactions, making it difficult to separate structures by comparing intensity values among tissues. Instead, doctors rely on anatomical knowledge and prior observations and initially guide themselves based on tissue intersections with a high reflection. Distinct borders can be understood as clear edges in the ultrasound image, while completed borders refer to the doctor’s skill to fill the gaps in order to get anatomically correct smooth structures, looking at previous frames, for example.

**Fig. 2:**
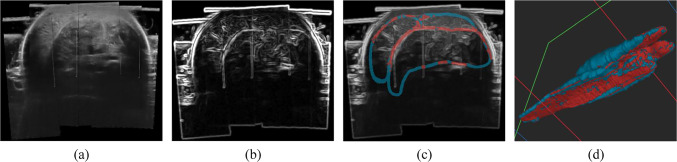
Distinct (red) and completed (blue) borders. **a** Ultrasound image, **b** Edges on the image, **c** 2D cross-sectional view with borders, and **d** 3D view of the borders

**Fig. 3:**
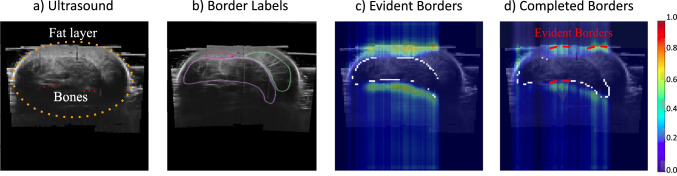
Seg-Grad-CAM activations for UNet for just the soleus muscle after split into distinct and completed borders: Labels correspond to purple: soleus, green: Gastrocnemius Lateralis, and pink: Gastrocnemius Medialis

We propose to calculate the distinct borders as the Hadamard product between the border label (*B*, performed by an expert, Fig. 3b) and the edged-smoothed ultrasound image created by applying smoothed and gradient filters to the ultrasound (Fig. 2b). The distinct Border ($B_{\textrm{distinct}}$) can be expressed as:

1 $$\begin{aligned} B_{\textrm{distinct}} = \textbf{Thres}(I *K_{\text {smooth}} *K_{\text {sobel}}) \odot B \end{aligned}$$

Figure 2 presents the challenging ultrasound images of the leg in a cross-sectional view (a) and the extracted edges (b). The obtained distinct borders are in red and the completed in blue (c). We can observe in the 3D view (d) the high probability of getting completed borders in deeper regions where the ultrasound signal is low.

*Pre-evaluated Seg-Grad-CAM*: The main objective of our method is to evaluate the ability of the network to delineate the borders, as physicians do. For this, we visualize the Seg-Grad-CAM of the completed and distinct borders. We observe the activation areas for specific pixels in order to understand the decisions taken by the networks. The core of the Seg-Grad-CAM is the gradient computations. The localization map $L^c_{\text {Seg-Grad-CAM}}$ is computed by first obtaining the gradient with respect to a specific pixel’s score class $Y^c$ in the output segmentation map (for $Y^c \in \{B_{\textrm{distinct}}, B_{\textrm{completed}}\}$), then computing the global average pooling of these gradients, and finally combining these with the activation maps and applying a ReLU activation. This process is represented by:

2 $$\begin{aligned} L^c_{\text {Seg-Grad-CAM}} \!=\! \text {ReLU}\!\left( \sum _{k} \!\left( \frac{1}{w \!\times \! h}\! \sum _{i} \sum _{j}\! \frac{\partial Y^c_{i,j}}{\partial A^f_{i,j}} \right) A^f \!\right) \!,\nonumber \\ \end{aligned}$$

where $A^f$ is the activation map of the $f\textrm{th}$ feature map from the last convolutional layer $L$ of the segmentation network when processing image $X$, being $\frac{1}{w \times h}$ the global average pooling operator, and $(i,j)$ the spatial coordinates in the feature map. Figure 3 presents the Seg-Grad-CAM for $B_{\textrm{distinct}}$ (c) and $B_{\textrm{completed}}$ (d) for just soleus muscle. We can observe that the border segmentation of one muscle takes into consideration the other muscles; this means that there exist a relationship between muscles which provides contextual information for segmentation. When segmenting distinct regions, we can observe an activation mainly in the distinct borders of all the muscles. For completed borders, activations appear in distinct borders of all the muscles, and additional regions like the bones, mimicking doctors’ procedure to use key points as reference.

## Experiments and results

In order to evaluate the accuracy of the networks to predict distinct and completed borders on ultrasound datasets, we train 3 different encoder–decoder architectures, using Monai framework [24] (an open-source Pytorch-based framework designed for medical imaging research), on 4 different datasets, for 3 different cross-validation runs. Losses like Dice, cross-entropy, and Dice-Cross-entropy were evaluated. To solve the imbalance problem in the thyroid and the leg dataset, the weighting of cross-entropy was calculated with the inverse of the percentage of pixels belonging to that class in the training set and normalization of the class weights to ensure they all sum up to 1. For our experiments, all datasets were split patient-wise in 70–20–10% for training, validation, and testing, respectively. Data augmentation techniques like flipping the images in the vertical axis and min-max histogram normalization were applied to the images. The network trained until convergence with batch size to 2. We did not use pre-trained weights due to the network’s adaptation on big size volumes, but in custom smaller datasets, we use that pre-trained weights can improve the network’s performance. We used Adam optimizer with a learning rate of 0.001 and a StepLR scheduler, with a learning rate decay frequency set at intervals of 10 epochs, coupled with a decay factor of 0.5. We evaluate the mean and standard deviation of different metrics, including Dice, Hausdorff distance 95% (HD95) and the normalized surface distance (NSD), for the images in the test dataset.

### Common border segmentation metrics evaluation

**Table 1:** Evaluated metrics with mean and standard deviation of the test datasets for 2D and 3D architectures

Dataset	Leg-3D-US			Nerve-UTP-2D		
Metrics	Dice	HD95	NSD	Dice	HD95	NSD
UNet	**0**.**789** ± **0**.**029**	**5**.**61** ± **1**.**62**	**0**.**962** ± **0**.**023**	**0**.**739** ± **0**.**283**	**11.28 **±** 15.62**	**0**.**815** ± **0**.**319**
A-UNet	0.587 ± 0.048	8.29 ± 1.58	0.866 ± 0.036	0.73 ± 0.298	11.72 ± 11.07	0.791 ± 0.316
UNeTR	0.519 ± 0.161	14.75 ± 4.12	0.757 ± 0.107	0.661 ± 0.249	18.06 ± 18.56	0.719 ± 0.258

Dataset	Thyroid			Prostate		
Metrics	Dice	HD95	NSD	Dice	HD95	NSD
UNet	0.888 ± 0.053	**5**.**12** ± **2**.**71**	0.966 ± 0.018	**0**.**816** ± **0**.**075**	6.84 ± 4.02	**0.952 **± **0**.**048**
A-UNet	**0**.**892** ± **0**.**033**	6.81 ± 1.67	**0.972 **± **0**.**014**	0.815 ± 0.092	**6**.**71** ± **4**.**05**	0.947 ± 0.065
UNeTR	0.636 ± 0.105	27.20 ± 8.12	0.766 ± 0.034	0.523 ± 0.179	10.66 ± 6.28	0.77 ± 0.217

With HD95 being the Hausdorff distance and NSD the normalize surface distance. In bold is highlighted the best performance

We calculate different segmentation metrics, including the normalized surface distance (NSD) that specifically evaluates the focus on the borders. We report the results in Table 1. Our findings indicate that the UNet transformer architecture consistently displayed inferior performance across all datasets compared to the other two network architectures, likely due to the number of data transformers’ architectures needed. It showed an average performance gap of 6% in 2D versus 27% in 3D. Selecting the best architecture between UNet and A-UNet was not straightforward due to their closer metric values, especially for the thyroid and prostate datasets. We performed additional evaluations to understand the dispersion of the metrics for each slice, by computing violin plots available in the supplementary material. We observe that the statistical difference depends on the metric used. In the prostate dataset, using the Dice metric does not present a difference between UNet and A-UNet, while using the NSD score presents a difference. The selection of the metric is important and depends on the modality and the desired clinical use.

**Fig. 4:**
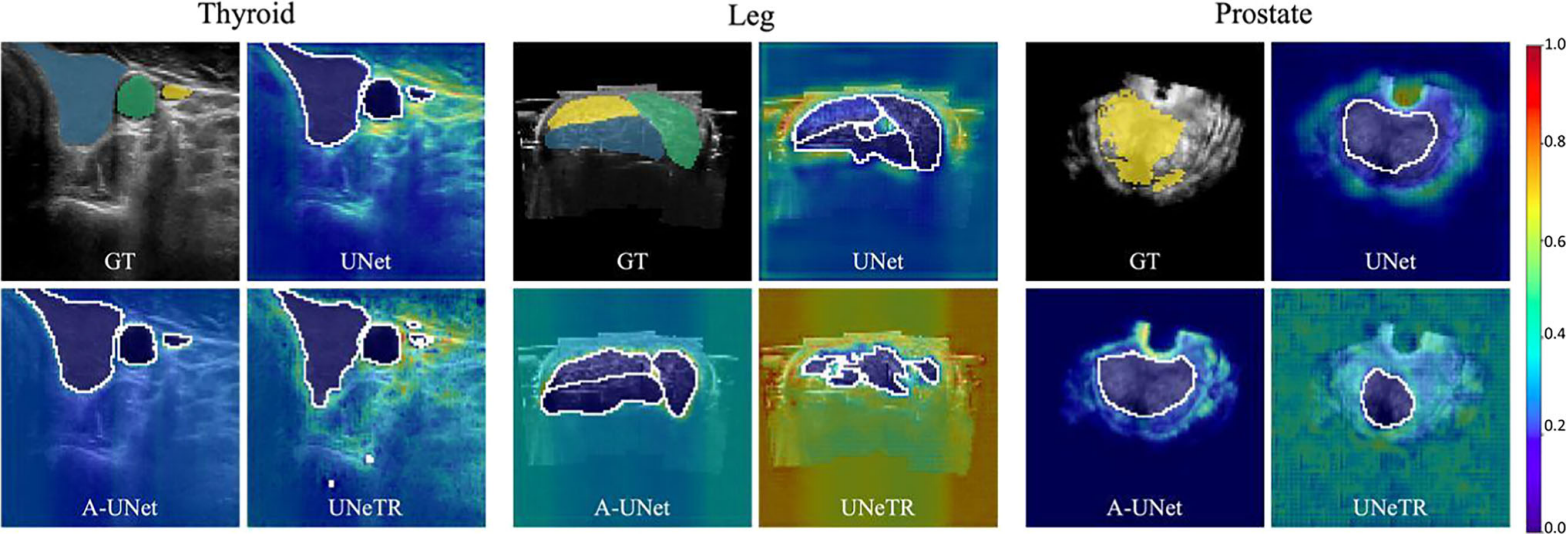
3D Seg-Grad-CAM evaluation of the background label with predictions border overlapped in white

Further, we compute the Seg-Grad-CAM of the background label in order to understand how the network delineates the borders of desired structures, see Fig. 4. To understand the influence of different attention modules on different architectures, we visualized the focus areas of the networks. In the case of UNet, Seg-Grad-CAM has wider spread attention over the image, while Attention UNet uses the attention modules to focus on more specific areas, and mainly the borders. Also, in the case of UNeTR, patch-wise processing of the image results in patch-wise Seg-Grad-CAM patterns. In fact, since ultrasound has no well-defined borders, the self-attention maintains active ineffective global correlations between patches. It can be observed that networks mainly focus on structures with high reflection, such as tissue interfaces, and depending on the network the attention area of the background gets slightly more dispersed or focused. However, it doesn’t focus on any specific anatomical points, and therefore, it does not provide sufficient information for analyzing the networks.

### Evaluation of network accuracy for predicting distinct and completed borders

**Fig. 5:**
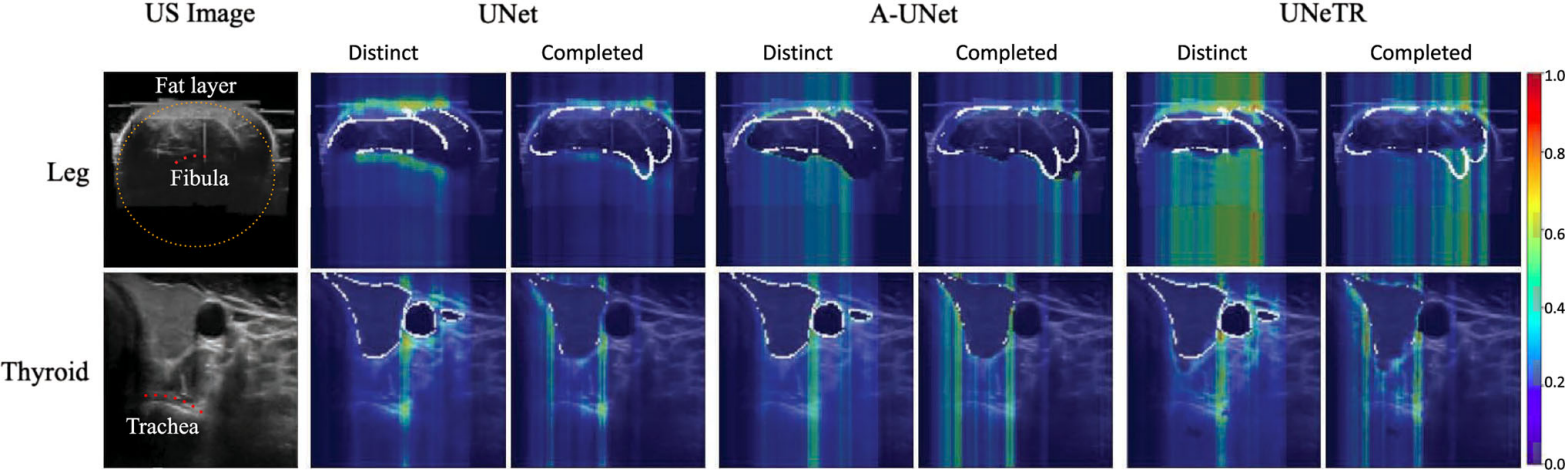
Anatomical networks observations: On the thyroid, we can identify the Trachea, while on the leg other structures like the Fat layer and the Fibula are also activated

In our approach, we evaluate instead the image areas activated using Seg-Grad-CAM for distinct and completed borders (see Fig. 5). We observe that the attention areas are different depending on the network architectures. In general, the distinct borders get activated in interfaces with high reflection. In the case of leg muscle segmentation, there is a high attention on the fat layer outlining the upper part of the muscle, resulting in a clearer border.

In the case of completed borders, predictions get activated depending on the network attention method. For instance, UNetR divides the volume into patches, which worsens the completion of areas. UNet, on the other hand, handles features at different scales with the encoder, allowing it to take into account distinct borders when predicting completed ones. Additionally, we observe that other structures or organs, such as bones or trachea, were used to provide more context and improve the completed borders. This is similar to the way doctors look for key points to help them segment borders. We hypothesize that the striping artifacts could be due to the observation of the comet-tail artifact generated by the vein in the thyroid dataset or due to the compounding lines created when compounding several 2D B-mode images in the leg dataset. However, more research should be done in this direction.

**Table 2:** True-positive percentage in the test dataset predicted with respect to the complete border

	Leg-3D-US			Thyroid		
	$B_{\text {TP}}$	$B_{\text {Distinct}}$	$B_{\text {Completed}}$	$B_{\text {TP}}$	$B_{\text {Distinct}}$	$B_{\text {Completed}}$
$\textrm{Reference}_{\textrm{Border}} [\%]$	100	$54.8 \pm 7.6$	$45.2 \pm 4.3$	100	$86.7 \pm 4.8$	$13.3 \pm 4.8$
$\textrm{UNet}_{\textrm{Predictions}}$	$82.1 \pm 3.3$	$42.2 \pm 1.9$	$39.9 \pm 2.1$	$86.3 \pm 1.5$	$80.4 \pm 2.7$	$5.9 \pm 1.4$
$\mathrm{A-UNet}_{\textrm{Predictions}}$	$75.5 \pm 7.4$	$50.4 \pm 2.4$	$35.1 \pm 2.8$	$88.4 \pm 2.3$	$83.0 \pm 1.2$	$5.1 \pm 1.3$
$\textrm{UNetR}_{\textrm{Predictions}}$	$37.2 \pm 3.6$	$23.6 \pm 2.5$	$13.6 \pm 1.3$	$72.8 \pm 7.9$	$70.5 \pm 3.3$	$4.3 \pm 2.1$

Reference expresses the percentage of distinct and completed borders with respect to the total border

In a quantitative evaluation of distinct and completed prediction, we extract the borders of the ground truth labels and use this as a reference border. To obtain the percentage of distinct borders, we first calculate the Hadamard product, see Sect. 4 and then multiply it with the reference border. The completed border is the complementary of distinct, both summing 100% for the reference segmentation.

Table 2 provides an analysis of the leg and thyroid ultrasound datasets. For both datasets, the first row indicates the reference border percentage. The subsequent rows showcase predictions from three different segmentation models (UNet, A-UNet, and UNetR) in comparison to the total reference label border. Each model’s predictions are divided into three categories: total true positive ($B_{\text {TP}}$), distinct borders ($B_{\text {Distinct}}$), and completed borders ($B_{\text {Completed}}$), with the percentages and standard deviations provided. A high percentage for the prediction of distinct borders for all tasks can be observed, showing that the networks perform better for this task.

## Discussion and perspectives

In this work, we focus on analyzing ultrasound segmentation methods on their ability to learn boundaries between organs or structures. Different from MRI and CT scans, where similar tissues typically exhibit uniform values; in ultrasounds, intensities in the same tissue can fluctuate. We introduce a clinically inspired analysis that emphasizes border differentiation, categorizing them into distinct and completed borders. Seg-Grad-CAM is primarily employed in our study to enhance model interpretability and examine the network’s focal points but is not the central focus. Our findings reveal that these networks, much like medical professionals, adjust their focus depending on border clarity. The completion of borders takes into consideration both the clear borders and key anatomical points, alongside an intrinsically learned shape model.

However, it is important to clarify that Grad-CAM is subject to limitations that may lead to oversimplification, although it is widely used for its ability to visualize deep neural network decisions. Our experiment in supplementary material, including the evaluation of “signal lost patches” for the leg dataset and the impact of noise and contrast artifacts, evaluates the Grad-CAM’s robustness and its capacity to ignore non-informative areas while effectively highlighting relevant features. Our evaluation in distinct and completed borders is independent of the explainability method; future works could explore the advantages of using other explainability methods more robust to noise.

A further contribution is the provision of an open-access 3D leg ultrasound dataset. Public labeled datasets add value to the biomedical community helping in advancing of medical diagnostics. The leg dataset depicts the anatomical intricacies of the lower limb musculature, which can be useful in improving therapeutic interventions, particularly in the context of muscular pathologies like Duchenne muscular dystrophy. Furthermore, the dataset’s utility extends to the field of sports medicine, where it can serve to analyze the relationship between an athlete’s performance and the dynamic evolution of leg muscles, enabling the refinement of personalized training regimens. Importantly, this initiative circumvents the constraints posed by conventional imaging modalities such as MRI and CT, offering a non-invasive, cost-effective, and repeatedly applicable alternative.

For quantitative evaluation, we calculate separately the performance of the network to learn distinct borders or more complex ones (completed). Such metric splitting can be done for other metrics like IoU or normalized surface distance to get an idea of individual activation depending on the complexity of the task. Future perspectives can explore how anatomical shape models are learned in the networks and boost their performance using the split in distinct and completed boundaries in the loss.

## Supplementary Information

Below is the link to the electronic supplementary material.

Supplementary file 1 (pdf 1794 KB)

## Data Availability

Data, code and/or material availability. Available at https://github.com/Al3xand1a/segmentation-border-analysis.

## Footnotes

1 ImFusion GmbH, Munich, Germany.
