# Supplementary material for: Ultrasound segmentation analysis via distinct and completed anatomical borders
Source: Int J Comput Assist Radiol Surg. 2024 May 25;19(7):1419–27. doi: 10.1007/s11548-024-03170-7 (PMC11588783; doi:10.1007/s11548-024-03170-7)
Supplement: Supplementary file 1 — (pdf 1794 KB) [file 11548_2024_3170_MOESM1_ESM.pdf]

Supplementary material of Ultrasound Segmentation Analysis via Distinct and Completed Anatomical Borders

Vanessa Gonzalez Duque[1,2,3,4*†], Alexandra Marquardt[1,2†], Yordanka Velikova[1,2], Lilian Lacourpaille[5], Antoine Nordez[5], Marion Crouzier[5], Hong Joo Lee[1], Diana Mateus[4], Nassir Navab[1,2,3]

[1*]Computer-Aided Medical Procedure and Augmented Reality (CAMP), CIT, Technical University of Munich,Garching bei Muenchen, Germany.
[2]Munich Center for Machine Learning, Munich, Germany.
[3]Munich Data Science Institute, Munich Germany.
[4]LS2N laboratory at Ecole Centrale Nantes, Nantes, France.
[5]MIP laboratory, EA 4334, F-44000 Nantes, France.

*Corresponding author(s). E-mail(s): vanessag.duque@tum.de;
[†]These authors contributed equally to this work.

This supplementary material includes a comprehensive overview of our qualitative and quantitative results, the limitations of the Seg-Grad-Cam explainability method, and further details on implementation.

1 Qualitative results

This study underscores the significance of comprehending the distinctions in ultrasound segmentation as opposed to CT or MRI segmentation. By adopting a novel approach to evaluate the issue—distinguishing between evident and interpolated border evaluation—we elucidate why more sophisticated models that excel with natural images fall short in ultrasound contexts. This insight is crucial for directing improvements in network designs, loss functions, and training methodologies. The focus of this research is not on identifying the superior networks or the statistical significance of the findings. Instead, it emphasizes the necessity of a unique evaluation framework for

ultrasound architecture. Our analysis of the architectures into evident and completed borders is a redefinition of the evaluation problem, which is independent of:

- the visualization method (Seg-Grad-Cam)
- the architectures (UNet, A-UNet, UNeTR)
- the loss function (Cross-entropy-loss, Dice)
- the metrics (Dice Score, Hausdorff distance, False Positives)

Figure 1 presents additional qualitative results complementary to Figure 3 in the main paper. It presents Seg-Grad-Cam activations of the 3 muscles together and for Gastrocnemius Medialis and Gastrocnemius Lateralis independently. Activations are divided into completed and distincted borders in blue and red respectly. Segmentation of all the muscles together take into account the fat layer of the leg and create smooth muscles that follow the shape of the leg. Independent segmentation of Gastrocnemius Medialis take into account the Fibula bone reference point. While Gastrocnemius Lateralis takes into account the Tibia bone. Such reference points are the ones used for clinicians in the delimitation of the structures.

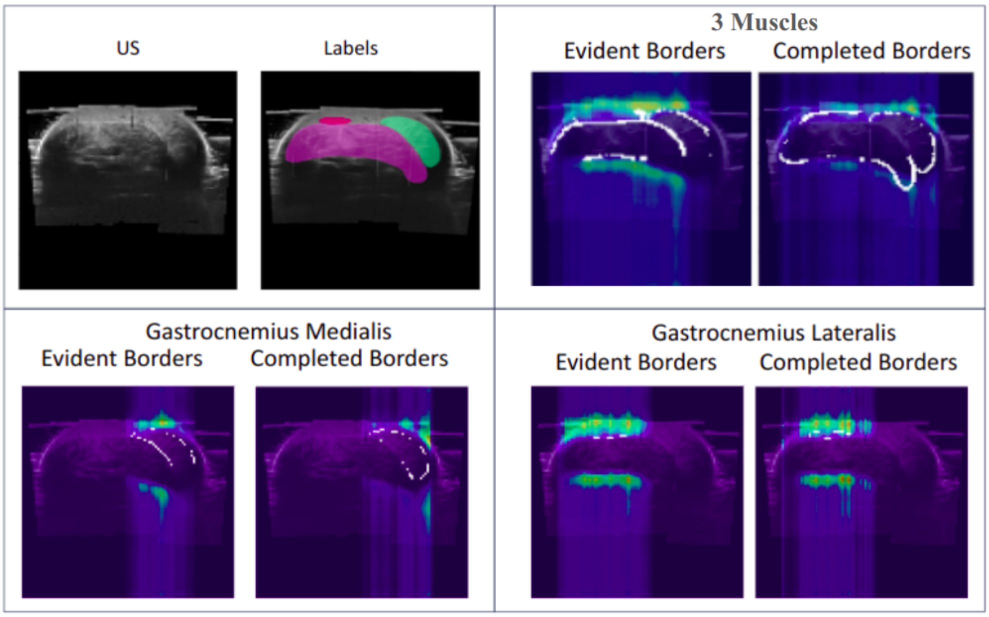

Fig. 1 Seg-Grad-Cam activations for UNet for completed borders in blue and distinct borders in red. Activations are presented per 3 muscles together and for GM and GL independently.

We evaluating the Seg-Grad-Cam activated areas for the thyroid in artifacts that Clinicians use to identify veins and arteries. See Figure 2. We observe that UNet and Attention UNet focus in the comet-tail artifact (in orange). However, just UNet focus on the acoustic enhancement area under the Jugular vein (in green) and additional features that consider important, reason why it struggle to classify such pixels and

perform less good than Attention UNet in terms of Dice and Normalize surface distance as can been seen in the violin plots 3 4. However we consider UNet to be closer to the clinical practice.

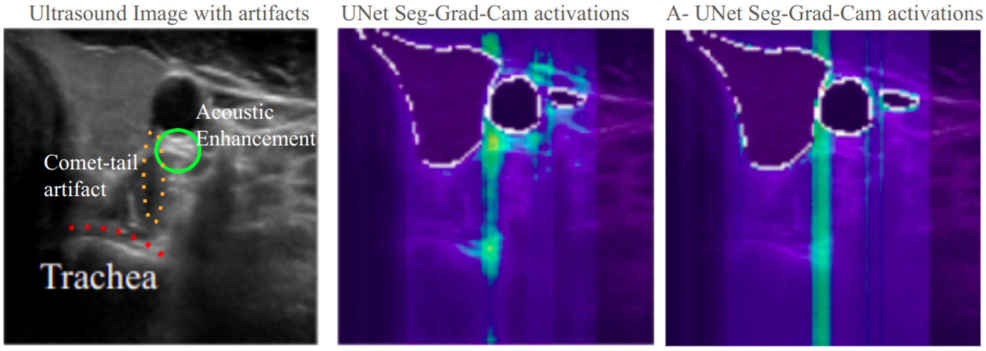

Fig. 2 Ultrasound image with artifacts and Seg-Grad-Cam activations for UNet and Attention UNet.

2 Quantitative results

We visualize the distribution of dice scores and the normalized surface distances (NSD) with violin plots.

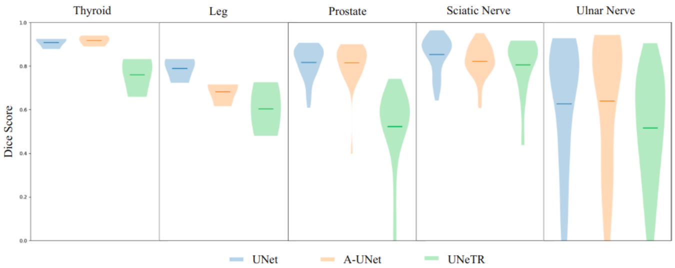

Fig. 3 Violin plots of Dice metric evaluated per slice on 3D volumes. UTP-nerve dataset contains 2 different groups of nerves that are evaluated independently.

Figures 3 and 4 present violin plots for the Dice Score and Normalized Surface Distance (NSD) across three distinct architectures over five datasets. In the prostate Dice Score analysis, no significant distinction is noted between U-Net and Attention U-Net, whereas a notable variance is observed in their NSD scores. This highlights the necessity of choosing metrics that reveal meaningful differences in evaluating the performance of architectural designs.

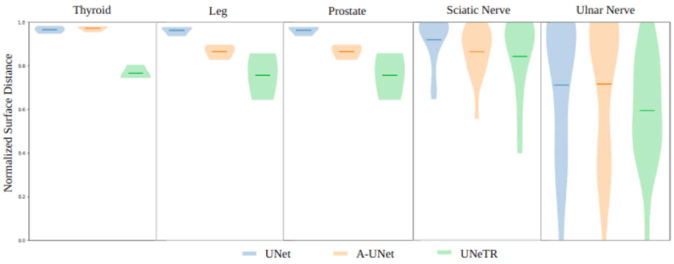

Fig. 4 Violin plots of Normalize Surface Dice metric evaluated per slice on 3D volumes. For Prostate-NSD the violin plots evidence a significant difference not observed for Dice score in Fig 7.

3 Seg-Grad-Cam limitations

The use of Seg-Grad-CAM in our research primarily aims to improve model interpretability, though it is not the main focus of our study. Despite its limitations [1], which can sometimes lead to oversimplification, Grad-CAM remains the preferred visualization technique for deep neural networks [2–7]. In line with previous research works, we utilize it to highlight activation in different architectures. Figure 5 presents the result of an experiment performed to evaluate the oversimplifications and robustness of the Grad-Cam to images with noise and contrast artifacts. We investigated potential oversimplifications, such as ”signal lost patches” where artifacts might falsely trigger activations for unrelated borders. Our findings suggest that absent signals do

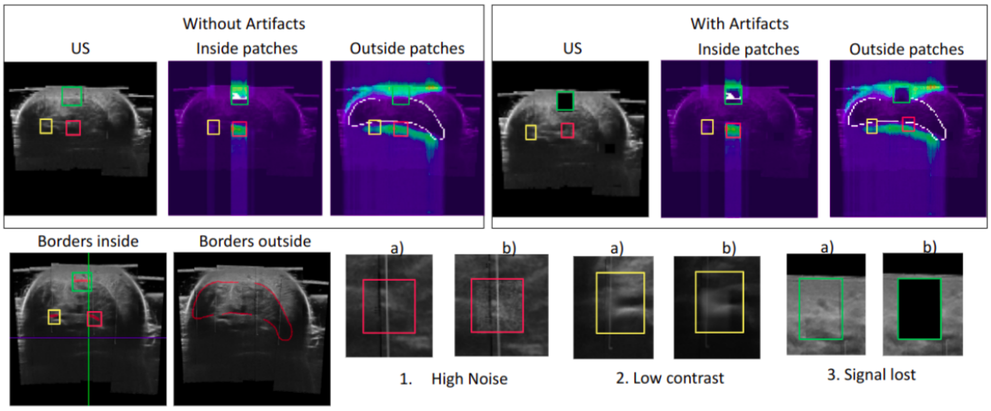

Fig. 5 Violin plots of Normalize Surface Dice metric evaluated per slice on 3D volumes. For Prostate-NSD the violin plots evidence a significant difference not observed for Dice score in Fig 7.

not lead to Grad-CAM activation's, underscoring the method's reliability in ignoring non-informative areas. Moreover, despite the presence of noise, evident borders within high-noise and low-contrast areas continue to present activation, elucidating Grad-CAM's effectiveness in identifying relevant features amidst interferences. We could think that for such kinds of artifacts, Grad-CAM is robust. This experiment

evaluates its utility in elucidating model focus, particularly valuable in ultrasound imaging for distinguishing between evident and completed borders, thereby enhancing the explainability of model decisions.

4 Implementations details

Using pretraining weights in the context of ultrasound volume segmentation is usually not feasible. In our case, since the volumes were big, we needed to adapt the size of the architecture, so we could not use the pre-trained weights. To address the imbalance problem, we use Cross-Entropy loss with labeling weighting of [0.19, 0.26, 0.31, 0.34] and [0.1, 0.25, 0.30, 0.31] for the Thyroid and the Leg dataset, corresponding to labels [background, Soleus, GM, GL] and [background, Thyroid, Aorta, Jugular] respectively. To find the weights, we calculate the inverse of the percentage of pixels belonging to that class in the training dataset and we normalize the class weights to ensure they all sum up to 1.
For A-UNet, we plot the attention gates in Figure 6, it contains the 8 attention maps of the last decoder step. We could observe that attention gates improve the sensitivity and accuracy of dense labels by suppressing feature activation in irrelevant regions, which explains why Seg-Grad-CAM of Attention UNet presents more focus activation maps, but the Figure did not give us information about the boundaries.

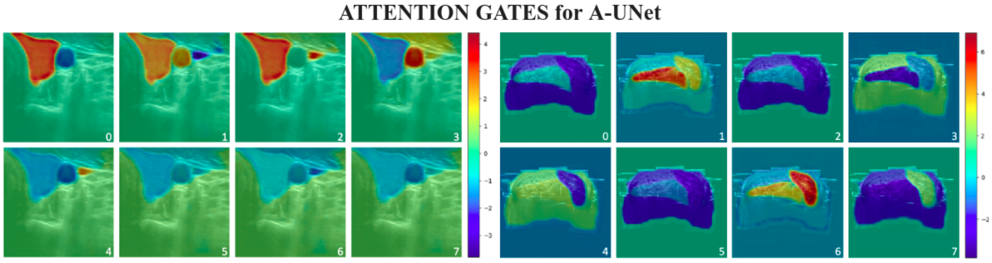

Fig. 6 Attention gates of Attention UNet for the Thyroid and the Leg dataset in the left and right side respectly.

References

[1] Chattopadhay, A., Sarkar, A., Howlader, P., Balasubramanian, V.N.: Grad-cam++: Generalized gradient-based visual explanations for deep convolutional networks. In: 2018 IEEE Winter Conference on Applications of Computer Vision (WACV), pp. 839–847 (2018). IEEE

[2] Hossain, A.A., Nisha, J.K., Johora, F.: Breast cancer classification from ultrasound images using vgg16 model based transfer learning. International Journal of Image, Graphics and Signal Processing **13**(1), 12 (2023)

[3] Xing, G., Miao, Z., Zheng, Y., Zhao, M.: A multi-task model for reliable classification of thyroid nodules in ultrasound images. Biomedical Engineering Letters, 1–11 (2023)

[4] Du, R., Chen, Y., Li, T., Shi, L., Fei, Z., Li, Y., et al.: Discrimination of breast cancer based on ultrasound images and convolutional neural network. Journal of oncology **2022** (2022)

[5] Jung, Y., Kim, T., Han, M.-R., Kim, S., Kim, G., Lee, S., Choi, Y.J.: Ovarian tumor diagnosis using deep convolutional neural networks and a denoising convolutional autoencoder. Scientific Reports **12**(1), 17024 (2022)

[6] Gunashekar, D.D., Bielak, L., Hägele, L., Oerther, B., Benndorf, M., Grosu, A.-L., Brox, T., Zamboglou, C., Bock, M.: Explainable ai for cnn-based prostate tumor segmentation in multi-parametric mri correlated to whole mount histopathology. Radiation Oncology **17**(1), 1–10 (2022)

[7] Ullah, I., Ali, F., Shah, B., El-Sappagh, S., Abuhmed, T., Park, S.H.: A deep learning based dual encoder–decoder framework for anatomical structure segmentation in chest x-ray images. Scientific Reports **13**(1), 791 (2023)
